# ‘There will be blood’^†^ A proof of concept for the role of haemorrhagic corpora lutea in the pathogenesis of endometriosis

**DOI:** 10.1093/hropen/hoae035

**Published:** 2024-05-29

**Authors:** Paolo Vercellini, Camilla Erminia Maria Merli, Paola Viganò

**Affiliations:** Department of Clinical Sciences and Community Health, Academic Centre for Research on Adenomyosis and Endometriosis, Università degli Studi di Milano, Milan, Italy; Department of Maternal and Child Health, Fondazione IRCCS Ca’ Granda Ospedale Maggiore Policlinico, Milan, Italy; Department of Maternal and Child Health, Fondazione IRCCS Ca’ Granda Ospedale Maggiore Policlinico, Milan, Italy; Department of Clinical Sciences and Community Health, Academic Centre for Research on Adenomyosis and Endometriosis, Università degli Studi di Milano, Milan, Italy; Department of Maternal and Child Health, Fondazione IRCCS Ca’ Granda Ospedale Maggiore Policlinico, Milan, Italy

## ‘Endometriosis—a disease because it is characterized by bleeding’. (Brosens, 1997)

In the 20th century, Ivo Brosens focused on the crucial issue of bleeding, which he considered to be at the core of the pathogenesis of endometriosis, with a simple, definitive, and catchy title (Brosens, 1997). Ivo had the rare gift of scientific synthesis, as when he defined the endometrioma as an extraovarian pseudocyst (Brosens *et al.*, 1996) or an extraovarian haematoma (Brosens *et al.*, 1994), or when he first drew attention to the then understudied, but highly risky, haemorrhagic consequence of deep infiltrating endometriosis (DIE), i.e. spontaneous haemoperitoneum in pregnancy (Brosens *et al.*, 2009).

Bleeding now returns as a *leitmotif* of the endometriosis trajectory and derailment, in the role of a potential precursor of DIE. In a study published in this issue of *Human Reproduction Open*, Chaggar *et al.* (2024) prospectively recruited and followed 51 premenopausal non-pregnant women who were consecutively evaluated for acute lower abdominal pain. Within 6 months of the acute episode, 7/15 (47%) women who presented with haemoperitoneum, i.e. the presence of blood clots and echogenic fluid in the peritoneal cavity, developed sonographic evidence of DIE, compared with 0/36 of those without haemoperitoneum. Interestingly, a haemorrhagic corpus luteum was identified as the origin of the intra-abdominal haemorrhage in 13/15 (87%) cases. As the authors are also well known for their exceptional expertise in ultrasonography, the possibility of a diagnostic bias can be reasonably ruled out.

None of the women in the haemoperitoneum group were using combined oral contraceptives or progestogen-only pills compared with 10/36 (28%) in the non-haemoperitoneum group. Three women in the first group were using a levonorgestrel-releasing intrauterine device (LNG-IUD). However, the LNG-IUD does not inhibit ovulation for more than a few months after insertion (Guillebaud, 2003). Thus, it is reasonable to assume that ovulation occurred in all 15 patients presenting with haemoperitoneum.

This cohort study follows the publication of a previous prospective pilot study by the same research group. Bean *et al.* (2019) followed 35 women with severe acute lower abdominal pain and detected *de novo* DIE at ultrasound follow-up in 4/6 (67%) of patients with evidence of haemoperitoneum compared with 1/29 (3%) of those without haemoperitoneum at baseline assessment. Seven of the eight women who presented with intra-abdominal bleeding had a haemorrhagic functional ovarian cyst. Two of these eight patients did not complete the follow-up.

Pooling data from the above studies, 11/21 (52%; 95% CI, 32–72%) patients presenting with haemoperitoneum-associated lower abdominal pain developed DIE within a few months of the acute episode compared with 1/65 (2%; 95% CI, 0.1–9%) of those without haemoperitoneum (relative risk, 34; 95% CI, 5–248). In addition, a bleeding functional ovarian cyst was the cause of haemoperitoneum in 19/23 (82.6%; 95% CI, 62–94%) patients with sonographic evaluation at presentation. To conclude, spontaneous haemoperitoneum not in pregnancy (SHniP) is very often associated with a haemorrhagic corpus luteum. Moreover, the association between SHniP and subsequent development of DIE is so strong that it can reasonably be considered causal.

The demonstration that substantial intraperitoneal bleeding is a precursor of infiltrating, fibrotic endometriotic lesions is extremely interesting and may open up a completely new view of the natural history of endometriosis. However, we should not mistake the finger for the moon here, because the most important information seems to be the central pathogenic role of haemorrhagic corpora lutea in the progression of endometriosis to advanced forms. In other words, the real precursor of DIE may not be haemoperitoneum *per se*, but what caused the haemoperitoneum, i.e. a haemorrhagic corpus luteum. If this is true, then haemoperitoneum acts as a common thread between the source of bleeding and the development of DIE, and what remains to be clarified are the biomolecular mechanisms along this pathway.

But there is a question positioned at an even higher pathogenic level: why is there such a high incidence of haemorrhagic corpora lutea in women who go on to develop DIE? The association between cystic corpora lutea and DIE was recently reported by the same research group also in a large prospective cohort study of more than 1000 women attending a general gynaecology clinic (Chaggar *et al.*, 2023).

The question may prove to be crucial, especially considering that haemorrhagic corpora lutea are the precursor not only of DIE, but also of many ovarian endometriomas. We followed 109 patients who did not use postoperative hormonal treatments for 2 years after surgical removal of endometriomas. A total of 27 (25%) participants developed a cyst recurrence, which in 11 (41%) cases was preceded by a haemorrhagic corpus luteum (Vercellini *et al.*, 2009). The central pathogenic role of ovulation in endometrioma formation is confirmed by the impressive protective effect of postoperative ovulation suppression after laparoscopic cyst removal (Zakhari *et al.*, 2021; Chiu *et al.*, 2022).

## Haemorrhagic corpora lutea, deep endometriosis, and endometriomas: who is the real culprit in this story?

The answer to the question of what causes such a high frequency of haemorrhagic corpora lutea, leading to the development of DIE and endometriomas through heavy bleeding, may lie in the nature of endometrioma formation.

Since the original study by Hughesdon (1957), it has been accepted that endometriomas are atypical cysts, in that they are ‘on’ the ovary rather than ‘in’ it, like most other cysts (e.g. serous, mucinous, and dermoid cysts). In other words, the ovary invaginates and duplicates so that the cortex itself constitutes the so-called pseudocapsule, which surrounds and contains the typical chocolate-like fluid consisting of old blood and siderophages (Brosens *et al.*, 1994, 1996). The first step in this process is the adhesion of the lateral gonadal aspect to the pelvic sidewall. Such adhesion is caused by inflammation originating from superficial endometriotic implants located on the peritoneum of the ovarian fossa and the posterior leaf of the broad ligament (Vercellini *et al.*, 2009). Indeed, according to Hughesdon (1957) and Brosens *et al.* (1994, 1996), superficial endometriotic implants are usually observed in correspondence with the site of ovarian inversion, whereas they are only irregularly found lining the inner wall of the pseudocyst.

The next question might be: ‘Where does the tarry, thick fluid content of endometriomas come from?’ The most plausible answer is not from the accumulation of menstrual debris secondary to the shedding and bleeding of endometriotic implants, but more likely from acute and heavy bleeding associated with ovulation. The strong relationship with haemorrhagic corpora lutea and the protective effect of ovulation suppression would be difficult to explain otherwise. When the ovary adheres to the pelvic side wall, blood from a corpus luteum is trapped and acts as an ‘invagination head’ causing ovarian duplication (Vercellini *et al.*, 2009).

If the establishment of peritoneal endometriosis on the pelvic sidewall is a prerequisite for endometrioma formation, a further question would be whether the superficial implants simply behave as innocent bystanders or may play an active role in ‘guiding’ the site of ovulation and also determining the haemorrhagic nature of what would otherwise be limited ovulatory bleeding.

## Does ovarian bleeding cause endometriosis or does endometriosis cause ovarian bleeding?

Ovulation involves inflammation and cytolysis. From a biomolecular perspective, superficial peritoneal implants adhering to the ovarian cortex may influence the site of ovulation through the surrounding secondary inflammatory microenvironment. Of relevance, cytokines such as interleukin-1 and -6, and tumour necrosis factor play a critical role in both endometriosis-associated inflammation and the ovulatory process (Vercellini *et al.*, 2014; Zondervan *et al.*, 2018; Kotlyar *et al.*, 2019). Thus, follicular rupture may occur causally, and non-casually, in correspondence with the site of superficial endometriotic implants.

Moreover, both endometrial and endometriotic cells release potent fibrinolytic molecules. Endometrial fibrinolytic activity effectively prevents intrauterine clot formation during normal menstrual flow. This also results in platelet deactivation and facilitates the transcervical expulsion of sloughed endometrium and blood (Davies and Kadir, 2012). Fibrinolysis also prevents scarring during endometrial repair. Indeed, the repeated tissue injury and repair process that occurs during the perimenstrual phase is uniquely characterized by a lack of scarring (Critchley and Maybin, 2011; Critchley *et al.*, 2020).

Fibrinolysis interferes with thrombus formation via the conversion of plasminogen to plasmin induced by tissue plasminogen activator (tPA) and urokinase plasminogen activator (uPA) enzymes. The endometrium is a rich source of tPA and uPA (Critchley and Maybin, 2011; Critchley *et al.*, 2020). Importantly, endometrial concentrations of plasminogen activators increase during the proliferative phase and peak at mid-cycle, i.e. just during the ovulatory phase (Davies and Kadir, 2012). Thus, in addition to driving the site of ovulation, superficial endometriotic implants may create a pro-haemorrhagic environment around the ovulatory stigma.

Higher levels of uPA have been observed in the endometrium of women with endometriosis. According to some researchers, this would increase the capacity of refluxed endometrial fragments to implant in the pelvis due to an increased potential to degrade the extracellular matrix (Zorio *et al.*, 2008). At the same time, this would also increase the local pro-haemorrhagic potential of endometrial fragments once ectopically implanted. Indeed, patients with endometriosis generally experience heavy menstrual bleeding (HMB) (Parazzini *et al.*, 2017; Shafrir *et al.*, 2018). The endometrium of women with HMB is characterized by a high production of proteolytic enzymes, such as matrix metalloproteinases, and fibrinolytic activity (Ferenczy, 2003), and synthesizes more vasodilatory PGE_2_ than vasoconstrictive PGF2α. In addition, the endometrium of women with HMB releases higher than normal amounts of prostacyclin, known to inhibit platelet aggregation and generate vasodilatation, and shows reduced expression of the potent vasoconstrictor endothelin-1 and increased expression of neural endopeptidase, its metabolizing enzyme (Livingstone and Fraser, 2002; Critchley and Maybin, 2011; Davies and Kadir, 2012; Critchley *et al.*, 2020; Jain *et al.*, 2022). A local decrease in vascular impedance may increase blood loss.

Thus, it can be further hypothesized that peri-ovarian superficial endometriotic implants, in addition to determining the site of ovulation, may also promote excessive bleeding, ultimately leading to the formation of a haemorrhagic corpus luteum.

## From micro- to macro-haemorrhagic endometriosis: corpus luteum bleeding as the origin of deep endometriosis and endometriomas

Chaggar and co-workers pointed out the possible presence of undiagnosed superficial peritoneal endometriosis as a limitation of their study, also because it is impossible to know ‘*how this could have contributed to the development of deep endometriosis*’. They also interpreted their findings as the result of peritoneal healing occurring over a blood clot, so that endometrial cells present in the peritoneal fluid, regardless of the presence of endometriosis, ‘*become trapped underneath the peritoneal surface and trigger the development of deep disease*’ (Chaggar *et al.*, 2024).

Although this is certainly possible, it cannot be excluded that no deep endometriosis would have developed in the absence of superficial implants, since according to the blood-based pathogenic hypothesis described above, ovarian haemorrhage from corpora lutea is provoked exactly by the local pro-haemorrhagic environment induced by peri-ovarian peritoneal endometriosis.

Supposedly, most women experience retrograde menstruation, some develop limited peritoneal implants, while only a minority eventually progress to advanced endometriosis. If the transtubal menstrual reflux theory is correct, the necessary condition for the onset of all endometriotic lesions is the co-presence of endometrial cells and blood. In light of the findings of Bean *et al.* (2019), Chaggar *et al.* (2024), and Vercellini *et al.* (2009), it appears that the amount of intra-pelvic blood makes the difference between the establishment of only superficial peritoneal lesions or the progression to deep lesions and endometriomas. In other words, superficial peritoneal endometriosis would be an early, micro-haemorrhagic form, because retrograde menstruation allows only a limited amount of blood to reach the pelvis, whereas deep lesions and endometriomas would be advanced, macro-haemorrhagic forms, because bleeding from a corpus luteum can lead to haemoperitoneum. If dense adhesions between the ovary and the pelvic sidewall prevent blood pouring from a corpus luteum from escaping, an endometrioma may form, whereas if blood can flow freely towards the most dependent part of the pelvis, a deep lesion may form. If the blood distribution is mixed, both types of lesions may develop simultaneously. In all cases, the blood, not the endometrium, would be the main pelvic aggressor.

Indeed, research efforts have focused more on the endometrial rather than on the blood component of retrograde menstruation. Nevertheless, several studies, notably by the group of Jacques Donnez, have demonstrated an iron overload in the pelvis of patients with endometriosis (Lousse *et al.*, 2009, 2012; Defrère *et al.*, 2011; Donnez *et al.*, 2016). In extreme synthesis, pelvic macrophages internalize refluxed erythrocytes and participate in the process of iron recycling. However, when an excess of blood overwhelms the scavenging capacity of pelvic macrophages, non-protein-bound, catalytic iron is released into the peritoneal fluid. This generates reactive oxygen species (ROS), which in turn damage the fragile mesothelial lining, expose the sub-mesothelial connective tissue, facilitate implantation of regurgitated endometrial fragments, and promote neo-angiogenesis and endometrial cell proliferation through various molecular and cellular factors (Ng *et al.*, 2020; Wyatt *et al.*, 2023; Ni and Li, 2024).

The above process may explain the development of superficial peritoneal endometriosis when a limited amount of blood is present in the pelvis (micro-haemorrhagic endometriosis). Chaggar *et al.* (2024) rightly suggest that the same mechanism is at work when haemoperitoneum develops because, in addition to causing oxidative stress-induced mesothelial lining breakdown of vast peritoneal areas, a large amount of blood leads to platelet activation and favours epithelial–mesenchymal transition and fibroblast–myofibroblast transdifferentiation. Indeed, the presence of clots is evidence that fibrinolysis is inadequate and that fibrin bridges are formed between adjacent structures. This is the beginning of an inter-organ adhesion process that leads to the burial of what were once superficial endometriotic implants, and to fibrogenesis, which is the hallmark of DIE (Guo 2018; Vigano *et al.*, 2018; Viganò *et al.*, 2020; Garcia Garcia *et al.*, 2023). Thus, what Chaggar *et al.* (2024) define as ‘*peritoneal healing over a blood clot*’ may instead be the result of adhesion between organs whose mesothelial covering has been damaged by catalytic iron-induced oxidative stress. For example, Douglas pouch DIE may be interpreted as ex-superficial peritoneal endometriosis buried by adhesions between the anterior rectal and posterior uterine aspects.

The mechanisms underlying mesothelial injury, post-traumatic peritoneal repair, the role of blood, ROS, fibrinolysis, platelet and macrophage activation, and fibroblast growth in driving the intra-abdominal adhesion formation and fibrotic process have been well reviewed by Koninckx *et al.* (2016). The fundamental role of intra-abdominal trauma in the pathogenesis of endometriosis has been repeatedly suggested by Canis *et al.* (2017 and 2020).

## Conclusions: is blood the *fil rouge* in the pathogenesis of all endometriotic lesion types?

**Figure 1.**
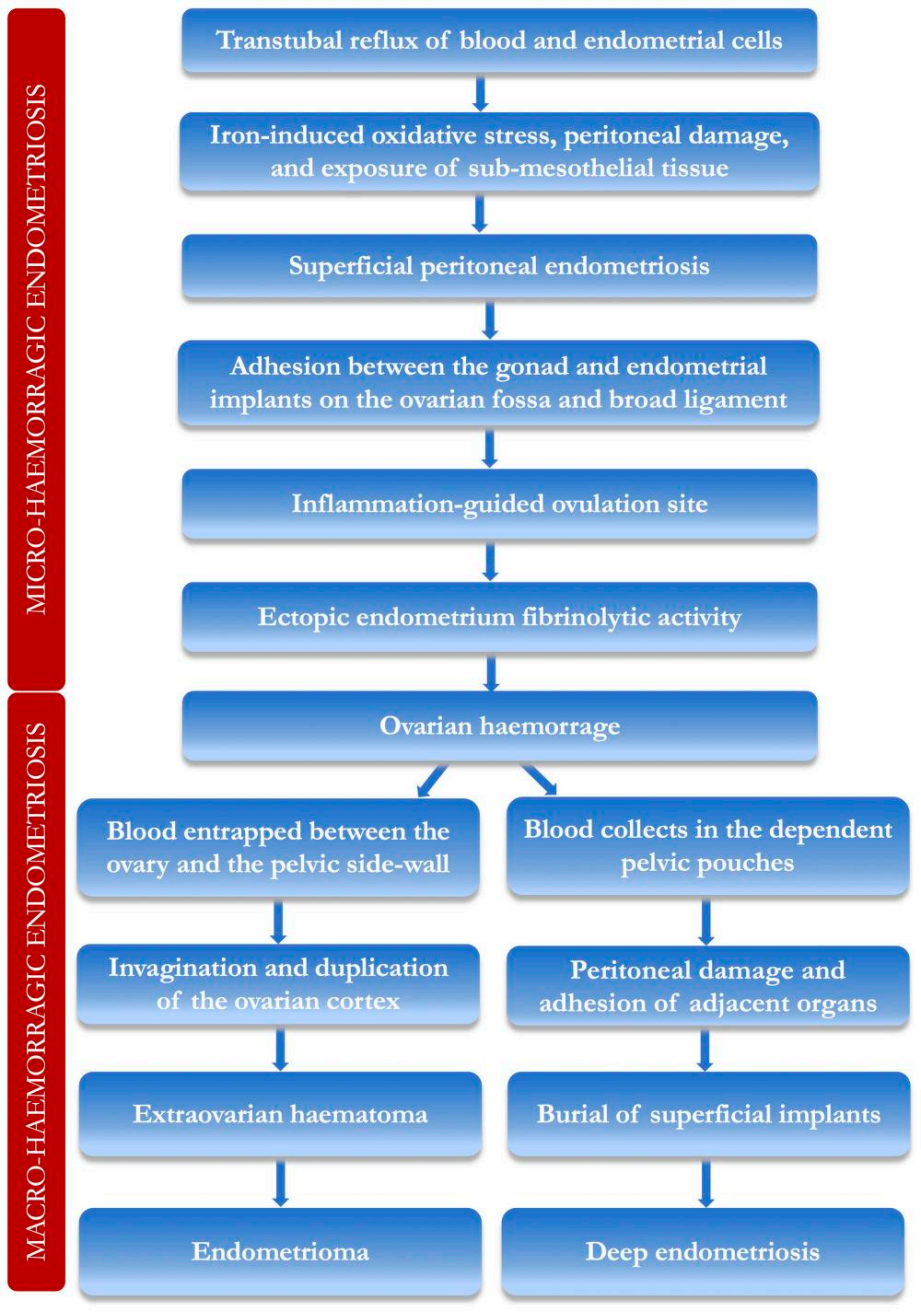
**A working hypothesis on the blood-based pathogenesis and pathophysiology of endometriosis.** The definition and progression of each step are based on information from Bean *et al.* (2019), Brosens *et al.* (1996), Canis *et al.* (2017), Chaggar *et al.* (2024), Defrère *et al.* (2011), Donnez *et al.* (2016), Garcia Garcia *et al.* (2023), Guo (2018), Jain *et al.* (2022), Koninckx *et al.* (2016), Hughesdon (1957), Lousse *et al.* (2009), Ng *et al.* (2020), Vercellini *et al.* (2009), Viganò *et al*. (2020), and Wyatt *et al.* (2023).

The working hypothesis of the blood-based pathogenesis of endometriosis is shown in Fig. 1. In synthesis, intraperitoneal blood may pave the way for the ectopic implantation of refluxed endometrial cells by inducing oxidative stress-mediated damage to the mesothelium. In this micro-haemorrhagic phase, the amount of blood is not sufficient to injure large areas of mesothelium, and indeed, extensive adhesions are not typically associated with limited superficial peritoneal endometriosis in the pelvis. However, because the lateral aspect of the ovary is juxtaposed to the pelvic sidewall exactly at the site of repeated monthly blood and endometrial efflux, the development of superficial lesions on the peritoneum of the ovarian fossa with secondary gonadal adhesion is common. The perilesional inflammation directs the rupture of the maturing follicle over the ectopic endometrium and creates a local pro-haemorrhagic environment that increases the risk of heavy bleeding from the corpus luteum.

In the case of blood entrapment, an endometrioma (extraovarian haematoma) may develop, whereas in the case of blood flowing freely towards the pelvis due to gravitational effects, clots will form and accumulate in the most dependent pelvic pouches, leading to DIE formation. This macro-haemorrhagic phase may be positively associated with the total amount of ovarian bleeding, which may therefore act as a rate-limiting step in the progression from early to extensive forms of endometriosis. Only a fraction of women with mostly undiagnosed superficial peritoneal endometriosis will develop the usually diagnosed endometriomas and infiltrating fibrotic lesions. This may partly explain the apparent discrepancy between the supposedly universal phenomenon of retrograde menstruation and the relatively low prevalence of diagnosed endometriosis. Most women may harbour micro-haemorrhagic endometriosis, albeit transiently, whereas few women would develop stable macro-haemorrhagic endometriosis.

Our pathogenic hypothesis is based on limited data, is highly speculative, does not consider many potential additional contributing causes, undoubtedly appears simplistic, and may not explain the onset of disease in many patients. There is no evidence on the real incidence of haemorrhagic corpora lutea in the general population and in the subgroup of individuals with established superficial peritoneal endometriosis, nor on the likelihood of developing deep lesions and endometriomas without transition through exposure to large amounts of blood. It is not possible to infer, even indirectly, the minimum volume of blood required to trigger the development of DIE and endometriomas, and it cannot be excluded that amounts smaller than those observed by Chaggar *et al.* (2024) may be enough to prompt the process. In other words, blood may act as the pivot of endometriosis progression even in the absence of easily identifiable moderate or severe haemoperitoneum causing acute lower abdominal pain.

What is now clear, thanks to the report by Chaggar and colleagues, is that refluxed endometrium alone is unlikely to be sufficient for the development of advanced endometriosis. The authors are to be commended for an extremely interesting and innovative study that may open a new window into the still-undefined pathogenesis of endometriosis. At the very least, the findings of Chaggar *et al.* (2024) provide a proof of concept that should now be confirmed by larger studies conducted by independent research groups. What better response to the ‘call for new theories’ proposed by the Endometriosis Initiative Group (2024)?

## Authors’ roles

P.Ve. conceived the study and drafted the original version of the article. C.E.M.M. and P.Vi. contributed to the acquisition of published information. All authors revised critically the drafts of the manuscript and approved its final version. All authors agree to be accountable for all aspects of the work.

## Notes

By Paul Thomas Anderson; produced by Ghoulardi Film Company and Scott Rudin Productions; distributed by Paramount Vantage and Miramax Films, USA, 2007.
